# Targeting FAM134B-DDX3X axis inhibiting AKT signaling in hepatocellular carcinoma

**DOI:** 10.1038/s41419-025-08080-3

**Published:** 2025-11-06

**Authors:** Jie Mo, Chen Su, Qiumeng Liu, Pengcheng Li, Lei Xu, Xin Long, Huifang Liang, Bixiang Zhang, Jin Chen

**Affiliations:** 1 Hepatic Surgery Center, Tongji Hospital, Tongji Medical College, Huazhong University of Science and Technology, Wuhan, Hubei 430030 P.R. China; 2 Department of Plastic and Cosmetic Surgery, Tongji Hospital, Tongji Medical College, Huazhong University of Science and Technology, Wuhan, Hubei 430030 P.R. China; 3 Department of General and Oncology Surgery, Wenchang People’s Hospital, Hainan, China

**Keywords:** Oncogenes, Targeted therapies

## Abstract

Family with sequence similarity 134, member B (FAM134B), known for its role as an ER-phagy receptor, has been implicated in the promotion of hepatocellular carcinoma (HCC) progression through the activation of the AKT signaling pathway. However, the precise mechanism underlying FAM134B’s activation of AKT signaling remains to be elucidated. This study aimed to investigate the interaction between FAM134B and DEAD-box helicase 3 X-linked (DDX3X) and its implications for HCC. We found that FAM134B interacts with DDX3X, preventing its proteasomal degradation by reducing K48-linked polyubiquitination and enhancing K63-linked polyubiquitination. This stabilization of DDX3X is crucial for AKT signaling activation, as DDX3X is known to promote the transcription of Rac Family Small GTPase 1 (Rac1), a key activator of the AKT pathway. Our results confirmed that FAM134B activates AKT signaling through the DDX3X-Rac1-AKT axis in HCC. Furthermore, we observed that DDX3X is upregulated in HCC and contributes to tumor progression. Interestingly, DDX3X not only activates AKT signaling but also increases FAM134B expression by enhancing its transcriptional activity, suggesting a positive feedback loop between these two proteins in HCC. Lastly, we explored the therapeutic potential of combining the DDX3X inhibitor RK-33 with FAM134B knockdown in HCC treatment. Our findings indicate that this synergistic approach may offer a promising strategy for HCC therapy.

## Introduction

Hepatocellular Carcinoma (HCC) is one of the most commonly diagnosed cancers globally [1], characterized by its complexity and multi-step, multi-genetic nature involving numerous genetic and epigenetic alterations. Despite its prevalence, the 5 year survival rate for advanced HCC is <2%, the lowest among solid tumors, with few patients benefiting from current treatments [2]. This underscores the urgent need for more effective therapeutic strategies.

Family with sequence similarity 134, member B (FAM134B), also known as RETREG1 or JK1, was initially identified as an oncogene in esophageal squamous cell carcinoma (ESCC) [3]. It is primarily recognized for its function as an endoplasmic reticulum autophagy (ER-phagy) receptor, facilitated by its reticulum homologous domain (RHD) and the LC3-interacting region (LIR) [4–7]. FAM134B’s role in various malignancies, including ESCC [3, 8], colorectal cancer (CRC) [9, 10], breast cancer [11, 12], and HCC [13, 14], is well-documented, with its function varying from an oncogene in ESCC and HCC to a tumor suppressor in CRC and breast cancer. Its dual role in cancer and the mechanisms behind it remain areas of active research. Evidence suggests that FAM134B may regulate cancer cell apoptosis through its ER-phagy receptor function [4]. Our previous research indicated that FAM134B is overexpressed in HCC tissues compared to adjacent non-tumor tissues and promotes HCC progression by activating the AKT signaling pathway [13], although the precise mechanism of this activation is yet to be fully elucidated.

DEAD-box helicase 3 X-linked (DDX3X), also known as DBX or DDX3, is a member of the DEAD-box helicase family, which plays a crucial role in RNA metabolism across various eukaryotic processes [15]. Located on the X chromosome at p11.3–11.23, DDX3X escapes X-inactivation in females and is expressed in all human tissues, participating in RNA transcription [16], pre-mRNA splicing [17], RNA export [18], and translation [19]. Its involvement in cancer biology has been extensively studied, with evidence suggesting that DDX3X promotes tumor progression in over ten types of cancers [20–29]. In HCC, studies have reported conflicting roles for DDX3X, with some indicating it is downregulated and inhibits cell proliferation and apoptosis resistance [27, 30, 31], while others suggest it is upregulated and promotes HCC metastasis [32]. Additionally, DDX3X has been implicated in the NLRP3 inflammasome, which inhibits ERK1/2 phosphorylation, enhancing SIRT7-mediated sorafenib resistance [33]. SIRT7 deacetylates DDX3X at K55, reducing its ubiquitination level [33].

In our study, we confirmed that FAM134B interacts with DDX3X, stabilizing the DDX3X protein by inhibiting its K48-linked polyubiquitination. Furthermore, we found that FAM134B activates the AKT signaling pathway through DDX3X-mediated Rac1 translation. Our research also confirmed the overexpression of DDX3X in HCC and its role in promoting HCC progression. Finally, we evaluated the therapeutic potential of the DDX3X inhibitor RK-33 in an HTVi model.

## Results

### FAM134B interacts with DDX3X

**Fig. 1:**
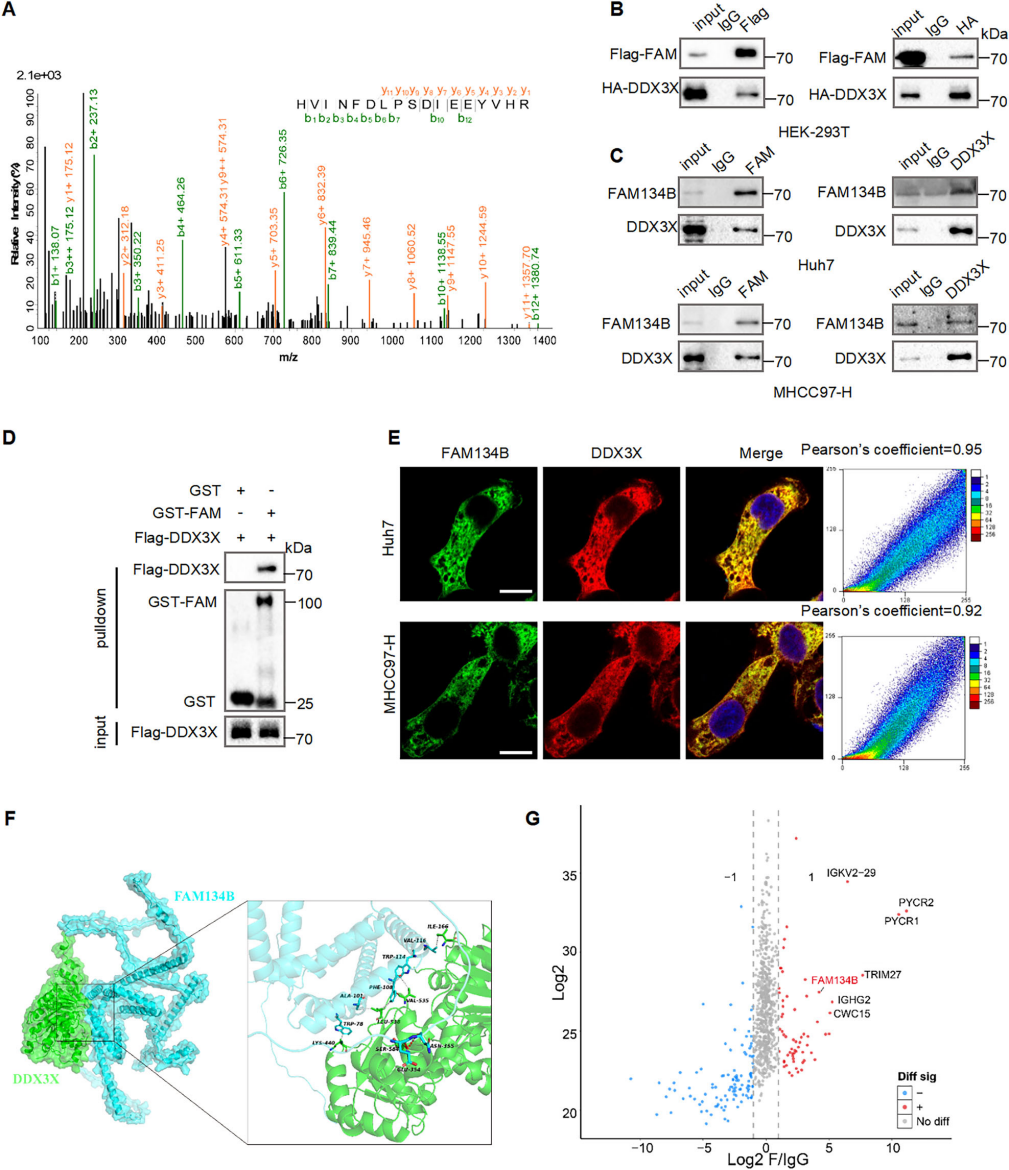
FAM134B interacts with DDX3X. **A** Peptides of DDX3X identified by FAM134B IP-MS. **B** HEK-293T cells were transfected with Flag-FAM134B and HA-DDX3X. Twenty-four hours later, cells were lysed with IP-lysis, then immunoprecipitated with control IgG, anti-Flag or anti-HA antibodies. Western blot detected the input and immunoprecipitates. **C** Huh7 and MHCC97-H cells were lysed with IP-lysis, followed by immunoprecipitation with either control IgG, anti-FAM134B, or anti-DDX3X antibodies. Western blot analysis was then performed to detect both the input and the immunoprecipitates. **D** Purified Flag-DDX3X protein was incubated with purified GST, GST-FAM134B and immobilized on beads. Affinity-isolated samples (top) and 5% of input DDX3X were analyzed by western blot (bottom). **E** Huh7 and MHCC97-H cells were fixed and incubated with anti-FAM134B and anti-DDX3X antibodies. Colocalization between FAM134B and DDX3X were examined by confocal microscopy. Scale bars, 10 µm. **F** Schematic diagram of FAM134B interacted with DDX3X. **G** Volcano plot of DDX3X binding proteins.

Our previous study established that FAM134B promotes hepatocellular carcinoma (HCC) tumorigenesis and metastasis by activating the AKT signaling pathway [13]. To elucidate the specific mechanism by which FAM134B activates AKT signaling, we conducted immunoprecipitation followed by mass spectrometry (IP-MS) to identify FAM134B binding proteins in HEK-293T cells. Among the enriched proteins, we focused on the DEAD-box helicase family protein DDX3X (Fig. 1A, Table 1), which has been reported to activate AKT signaling [32, 34–36]. The interaction between FAM134B and DDX3X was confirmed using exogenous co-immunoprecipitation (co-IP) assays (Fig. 1B). Endogenous co-IP further confirmed the interaction between FAM134B and DDX3X in Huh7 and MHCC97-H cells (Fig. 1C). Furthermore, the result of GST-pulldown assay indicated a direct interaction between FAM134B and DDX3X (Fig. 1D). Moreover, immunofluorescence (IF) confirmed the co-localization of FAM134B and DDX3X in the cytoplasm of HCC cells (Fig. 1E). Next, we used molecular docking analysis to investigate the interaction sites between the two proteins. Results showed that DDX3X and FAM134B primarily interacted through hydrophobic and hydrogen bonding interactions to form complexes. Specifically, the Glu354/Asn355 of FAM134B formed two hydrogen bonds with Ser584 of DDX3X, with additional stabilization through hydrophobic interactions (Fig. 1F, Table 2). Subsequently, we constructed plasmids with mutations in DDX3X (S584A) and FAM134B (E354A and N355A). Exogenous co-IP results showed that after the mutation of the hydrogen bond binding site, the binding between the two proteins was weakened (Fig. S1A), demonstrating the importance of these sites in the binding of the two proteins, further proved the molecular docking results. Then, we conducted IP-MS using Flag-DDX3X as a bait in HEK-293T cells. Results showed that DDX3X could also enriched FAM134B (Fig. 1G, Fig. S1B), further confirmed the interaction. Furthermore, Gene Ontology (GO) and Clusters of Orthologous Groups of proteins (COG) analyses of the enriched proteins revealed that DDX3X could enrich multiple proteins involved in various biological processes (Fig. S1C–E). Additionally, GO cellular component analysis indicated that DDX3X enriched a significant number of cytoplasmic and nuclear proteins (Fig. S1F). In summary, FAM134B interacts with DDX3X, and DDX3X exerts substantial biological effects.

**Table 1:** Peptide of DDX3X identified by FAM134B IP-MS.

Sequence	Unique	Position
HVINFDLPSDIEEYVHR	no	[512-528]
VGNLGLATSFFNER	yes	[535-548]
SPILVATAVAAR	no	[492-503]
DKDAYSSFGSR	no	[65–75]
ELAVQIYEEAR	no	[277-287]
HTMMFSATFPK	no	[377-387]
DREEALHQFR	no	[479-488]
VVWVEESDKR	yes	[419-428]
GKIGLDFCK	no	[334-342]
YLVLDEADR	no	[343-351]
FSGGFGAR	no	[593-600]
GLDISNVK	yes	[504-511]
SGFGKFER	yes	[114-121]
HAIPIIK	no	[209-215]
SSFFSDR	yes	[82–88]
YIPPHLR	no	[38–44]

**Table 2:** DDX3X and FAM134B interaction result.

Interaction	DDX3X	FAM134B	Distance (noH)
Hydrogen Bond	Ser584	Glu354	3.23
Hyrogen Bond	Ser584	Asn355	3.14
Hydrophobic Interaction	Lys440	Trp78	3.90
Hydrophobic Interaction	Leu538	Ala101	3.62
Hydrophobic Interaction	Leu538	Phe108	3.57
Hydrophobic Interaction	Val535	Trp114	3.66
Hydrophobic Interaction	Ile166	Val116	3.95

### FAM134B inhibits DDX3X proteasomal degradation

**Fig. 2:**
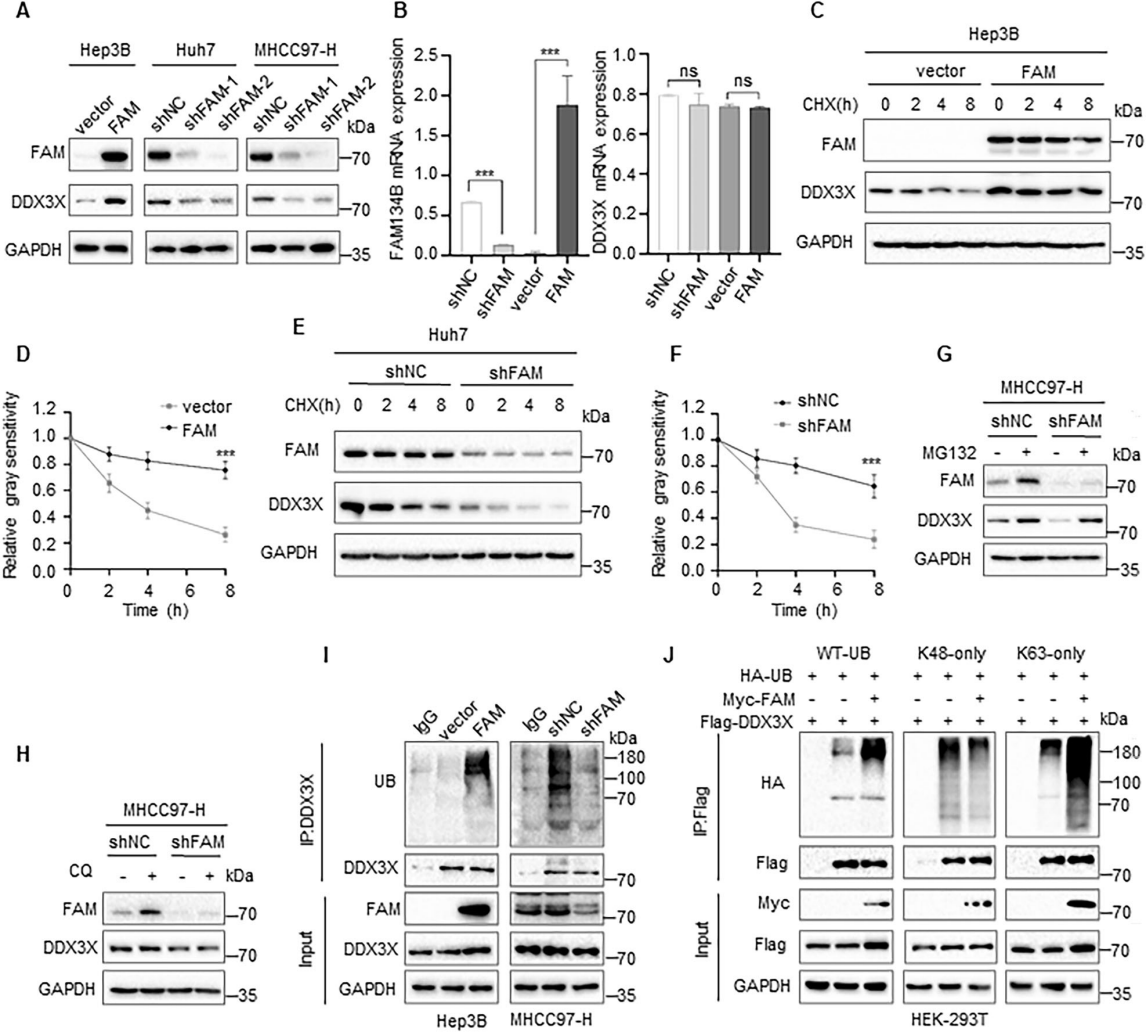
FAM134B inhibits DDX3X proteasomal degradation. **A** Western blot detected protein level of DDX3X in FAM134B overexpression or knockdown cell lines. **B** RT-qPCR detected DDX3X mRNA level in FAM134B overexpression (Hep3B) or knockdown (MHCC97-H) cell lines. **C**, **D** Hep3B cells stably expressing vector or FAM134B overexpression were treated with CHX (20 μM) for 0, 2, 4, 8 h. The degradation kinetics of DDX3X was monitored over time by Western blot. Data are mean ± SEM from three independent experiments, ***P < 0.001. **E**, **F** MHCC97-H cells stably expressing shNC or shFAM134B-1 were treated with CHX (20 μM) for 0, 2, 4, 8 h. The degradation kinetics of DDX3X was monitored over time by Western blot. Data are mean ± SEM from three independent experiments, ***P < 0.001. **G** MHCC97-H cells stably expressing shNC or shFAM134B-1 cells were treated with or without MG132 (10 μM, 4 h). Western blot detected DDX3X protein level. **H** MHCC97-H cells stably expressing shNC or shFAM134B-1 cells were treated with or without CQ (20 μM, 4 h). Western blot detected DDX3X protein level. **I** Hep3B cells stably expressing control or FAM134B, and MHCC97-H cells stably expressing control or shFAM134B-1, were collected using IP-lysis buffer. The cell lysates were then immunoprecipitated with either control IgG or anti-DDX3X antibodies. Both immunoprecipitates and input samples were analyzed by Western blot. **J** HEK-293T cells were transfected with Flag-DDX3X and HA-UB (WT, K48-only, K63-only), with or without Myc-FAM134B. After 24 h, cell lysates were immunoprecipitated with either control IgG or anti-Flag antibodies. Immunoprecipitates and input were analyzed by Western blot.

DDX3X is involved in the activation of the AKT pathway. Therefore, we hypothesized that FAM134B activates AKT by regulating DDX3X expression. Western blot analysis showed a positive correlation between FAM134B protein levels and DDX3X protein expression in FAM134B overexpression (Hep3B) or knockdown (Huh7, MHCC97-H) cell lines (Fig. 2A). RT-qPCR showed that the expression level of FAM134B had no effect on DDX3X mRNA level (Fig. 2B), suggesting the regulation might be occurred on the post-translational level. Treatment of FAM134B overexpressing or knockdown cells with cycloheximide (CHX) revealed that FAM134B stabilized DDX3X protein expression (Fig. 2C–F), suggesting post-translational regulation. Eukaryotic cells have two major protein degradation systems: the ubiquitin-proteasome system and the autophagy-lysosome system. Treatment of MHCC97-H cells stably expressing shNC or shFAM134B with proteasome inhibitor MG132 or lysosome inhibitor CQ showed that DDX3X expression was accumulated by MG132 (Fig. 2G) and was unaffected by CQ (Fig. 2H). Furthermore, the reduction in DDX3X due to FAM134B knockdown could only be restored by MG132 (Fig. 2G), confirming that FAM134B protects DDX3X from proteasomal degradation. We then examined the polyubiquitination level of DDX3X under conditions of FAM134B overexpression or knockdown. Interestingly, FAM134B expression was positively correlated with the total polyubiquitination level of DDX3X in Hep3B and MHCC97-H cells (Fig. 2I). Exogenous ubiquitination-based co-IP indicated that FAM134B slightly decreased DDX3X K48-polyubiquitination level but strongly increased DDX3X K63-polyubiquitination level (Fig. 2J) in HEK-293T cells, thus increasing the total polyubiquitination level (Fig. 2J). While K48-polyubiquitination is commonly associated with protein degradation, K63-polyubiquitination typically involves non-proteolytic functions [37]. However, emerging evidence supports a role for K63-polyubiquitination in stabilizing protein expression [38]. Therefore, our results confirmed that FAM134B stabilizes DDX3X protein expression by inhibiting its K48-polyubiquitination and increasing its K63-polyubiquitination.

### DDX3X promotes HCC proliferation and metastasis in vitro

**Fig. 3:**
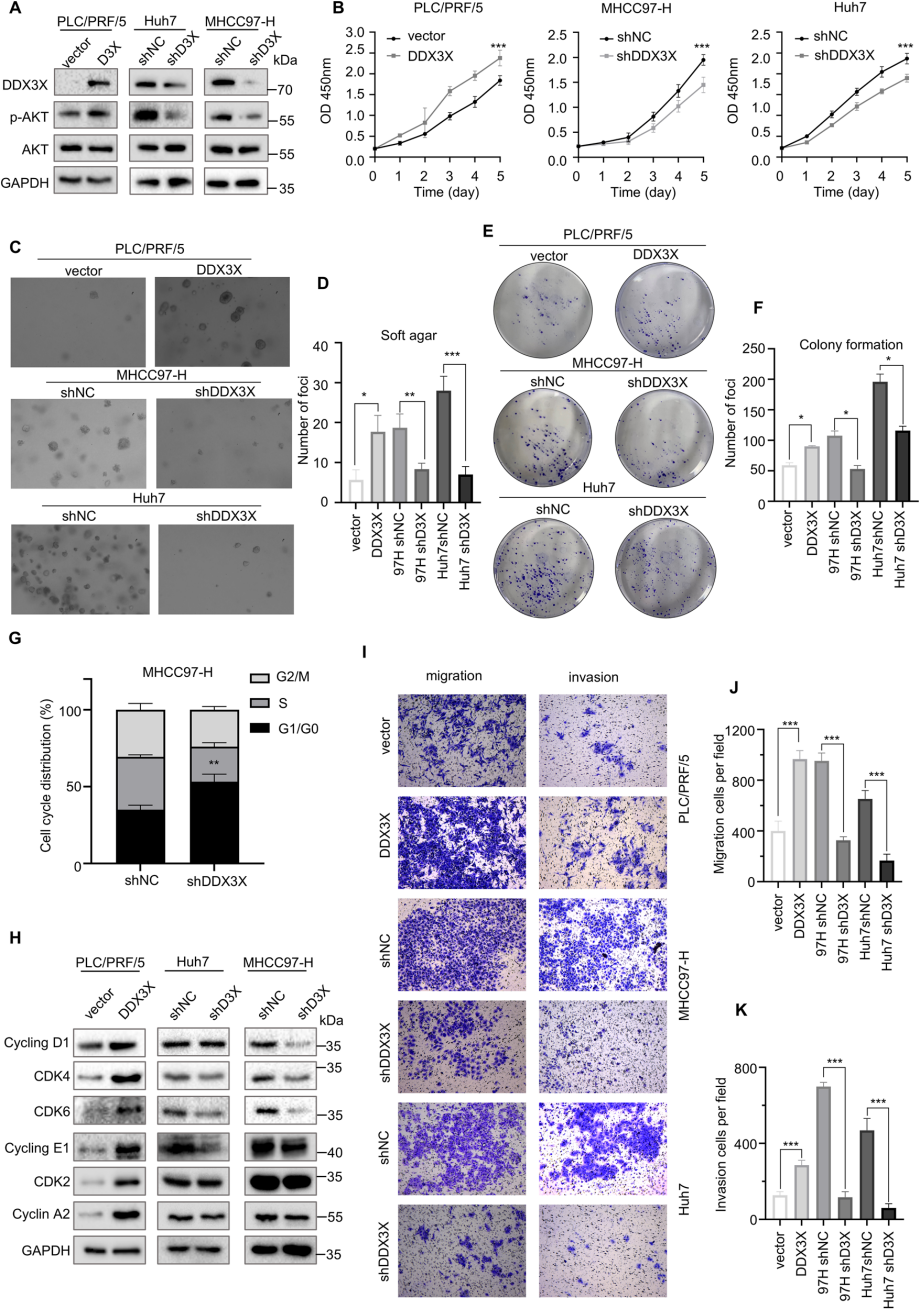
DDX3X promotes HCC proliferation, migration and invasion in vitro. **A** Western blot detected whether DDX3X overexpression and knockdown cell lines were successfully constructed, and detected the phosphorylated AKT (Ser473) level. **B**−**F** CCK-8 assay, *n* = 5 (**B**), soft agar assay, *n* = 3 (**C**, **D**) colony formation assay, *n* = 3 (**E**, **F**) detected the indicated cell line proliferation rate. Data are mean ± SEM, *P < 0.05, **P < 0.01, ***P < 0.001. **G** Flow cytometry tested the effect of DDX3X reduction on the cell cycle of MHCC97-H cells. Data are mean ± SEM from three independent experiments, **P < 0.01. **H** Western blot detected the expression of cell cycle related proteins in the indicated cells. **I**−**K** Transwell assay detected the effect of DDX3X on HCC cells migration and invasion. Data are mean ± SEM from at least three independent experiments, ***P < 0.001.

To investigate the role of DDX3X in HCC, we initially assessed DDX3X protein expression in various HCC cell lines. The results showed that DDX3X exhibited robust expression levels in the Huh7 and MHCC97-H cell lines, while its expression was minimal in the PLC/PRF/5 cell line (Fig. S2A). Subsequently, we established cell lines with either overexpression (PLC/PRF/5) or knockdown (Huh7, MHCC97-H) of DDX3X. Western blot analysis confirmed the successful construction of these cell lines (Fig. 3A). As expected, DDX3X overexpression increased the phosphorylation of AKT at Ser473, and knockdown of DDX3X decreased AKT Ser473 phosphorylation in HCC cell lines (Fig. 3A). Functional assays, including CCK-8 (Fig. 3B), soft agar (Fig. 3C, D), and colony formation assays (Fig. 3E, F), demonstrated that DDX3X overexpression enhanced HCC cell proliferation. Given DDX3X’s known association with the cell cycle [15], we performed flow cytometry analysis, which revealed that DDX3X depletion resulted in G1/G0 cell cycle arrest in the MHCC97-H cell line (Fig. 3G). Western blot analysis further indicated that DDX3X facilitated G1-S phase transition in MHCC97-H and Huh7 cells and promoted cell cycle progression across all phases in PLC/PRF/5 cells (Fig. 3H). Additionally, transwell assays (Fig. 3I–K) and wound healing assays (Fig. S2B–E) showed that DDX3X overexpression promoted HCC cell migration and invasion. Collectively, these in vitro results indicated that DDX3X significantly promotes HCC cell proliferation, migration, and invasion.

### DDX3X promotes HCC proliferation and metastasis in vivo

**Fig. 4:**
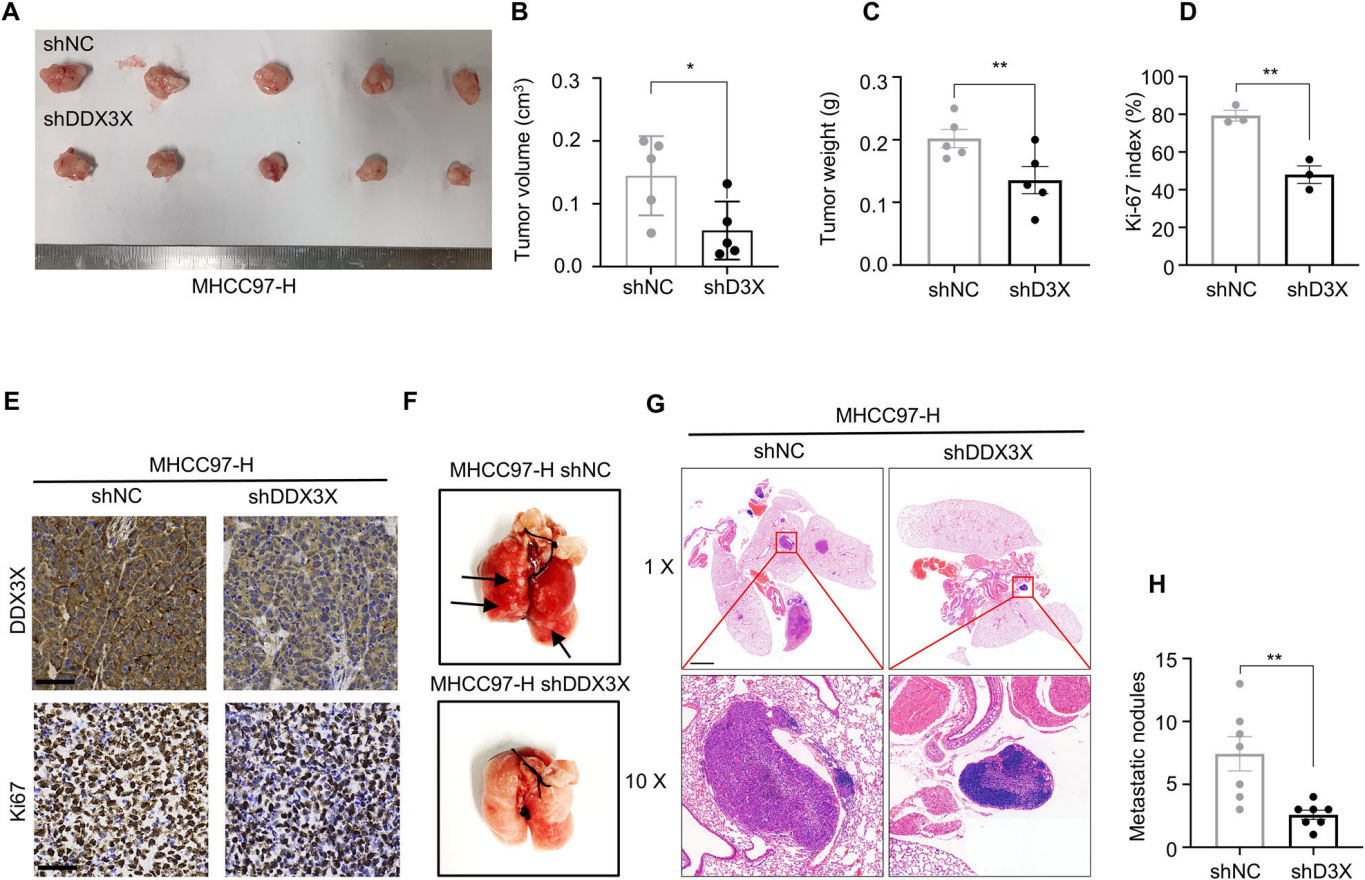
DDX3X promotes HCC proliferation and metastasis in vivo. **A**−**E** The gross image (**A**), tumor volume (**B**), tumor weight (**C**), Ki-67 index (**D**), IHC image (**E**) of subcutaneous tumor model from MHCC97-H shNC and MHCC97-H shDDX3X group, *n* = 5. Scale bar 100 μm. Data are mean ± SEM, *P < 0.05, **P < 0.01. **F**, **G** The gross image (**F**), H&E image of lungs (**G**), metastatic modules of lungs (**H**) of teil vein injection lung metastasis model from MHCC97-H shNC and MHCC97-H shDDX3X group, *n* = 7. Scale bar 2000 μm. Data are mean ± SEM, **P < 0.01.

Subsequently, we explored the in vivo role of DDX3X in HCC progression. In a subcutaneous tumor model, we observed that the knockdown of DDX3X in MHCC97-H cells resulted in a significant reduction in both tumor volume and weight (Fig. 4A–C). Additionally, the expression of Ki-67, a marker of cellular proliferation, was markedly lower in the DDX3X knockdown group compared to the control group (Fig. 4D, E). Furthermore, in a tail vein injection model designed to assess metastatic potential, we found that DDX3X knockdown in MHCC97-H cells led to a decrease in the number of metastatic nodules compared to the control group (Fig. 4F–H). Collectively, these in vivo findings underscored DDX3X’s oncogenic role in promoting HCC tumor growth and metastasis.

### FAM134B activates AKT signaling via DDX3X-mediated Rac1 translation

**Fig. 5:**
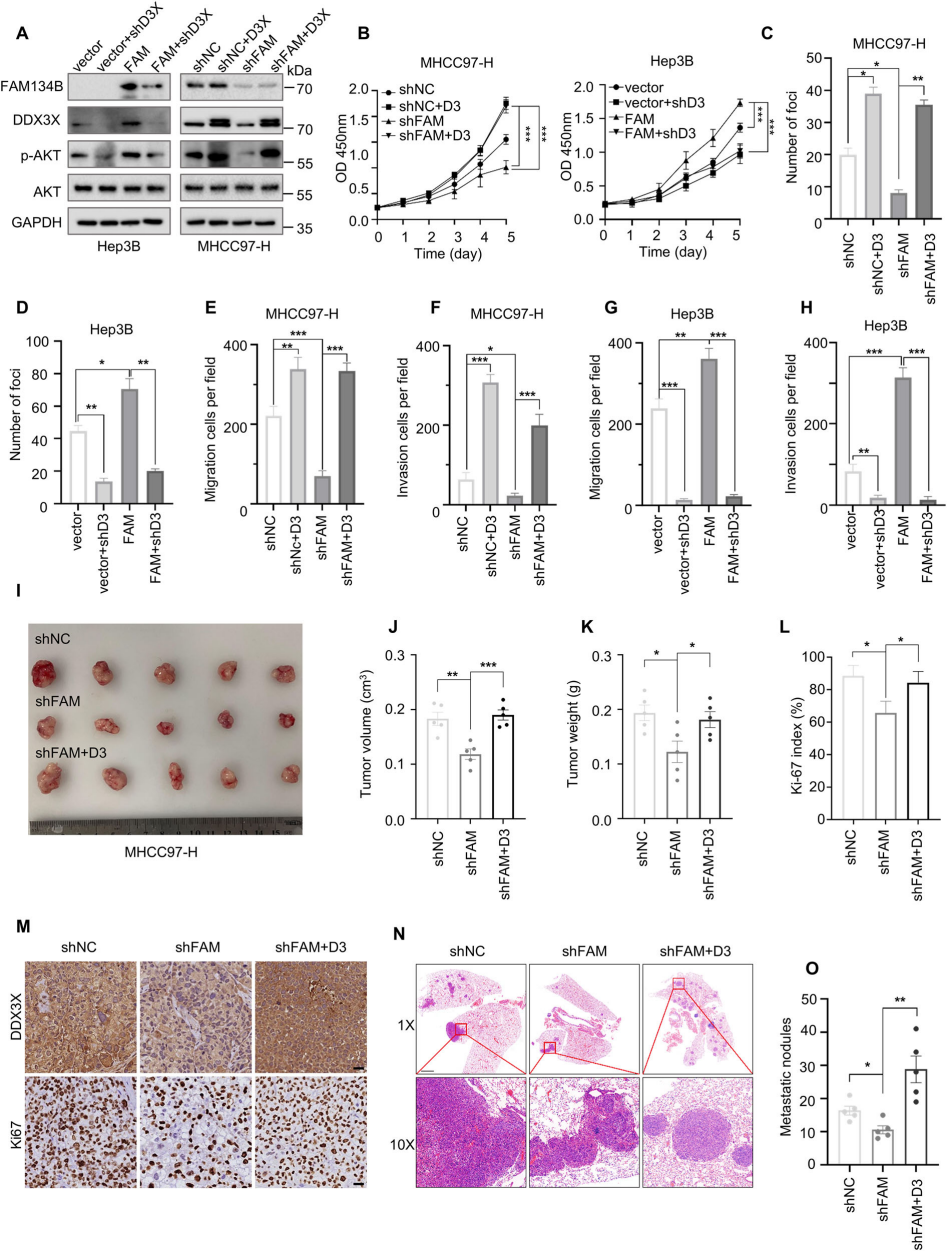
FAM134B promotes HCC progression via DDX3X. **A** Western blot detected the protein level of FAM134B, DDX3X, p-AKT (Ser473), AKT in rescue cell lines. **B**−**F** CCK-8 assay, *n* = 5 (**B**), colony formation assay, *n* = 3 (C and D) investigated whether the promotional effect of FAM134B on HCC proliferation was dependent on DDX3X. Data are mean ± SEM, *P < 0.05, **P < 0.01, ***P < 0.001. **E**−**H** Transwell assay investigated whether the promotional effect of FAM134B on HCC migration and invasion was dependent on DDX3X. Data are mean ± SEM from three independent experiments, *P < 0.05, **P < 0.01, ***P < 0.001. **I**−**M** The gross image (**I**), tumor volume (**J**), tumor weight (**K**), Ki-67 index (**L**), IHC image (M) of subcutaneous tumor model from shNC, shFAM134B-2 and shFAM134B-2 + DDX3X group, *n* = 5. Scale bar 100 μm, *P < 0.05, **P < 0.01, *** *P* < 0.001. **N**-**O** H&E image of lungs (**N**), metastatic modules of lungs (**O**) of teil vein injection lung metastasis model from shNC, shFAM134B-2 and shFAM134B-2 + DDX3X group. *n* = 5. Scale bar 2000 μm. Data are mean ± SEM, *P < 0.05, **P < 0.01.

Previous studies have demonstrated that DDX3X can activate the AKT signaling pathway through two mechanisms: (1) by activating the translation of Rac1, which has a structured 5’ UTR [34, 39, 40], and (2) by inhibiting the PTEN pathway [32, 35, 36]. Accordingly, we investigated whether DDX3X activates the AKT pathway through these two mechanisms in HCC. Western blot analysis revealed that the knockdown of DDX3X reduced Rac1 protein content (Fig. S3A), while having no effect on PTEN expression (Fig. S3A). Furthermore, wild-type (WT) DDX3X positively regulated Rac1 protein expression, whereas a helicase-defective DDX3X mutant (DDX3X^S382A,T384A^) had no effect on Rac1 expression (Fig. S3B), consistent with previous reports. Subsequently, we examined the expression of Rac1 and PTEN in MHCC97-H and Huh7 FAM134B knockdown cells. The results showed that the reduction of FAM134B had no effect on PTEN expression but inhibited Rac1 expression (Fig. S3C). Notably, Rac1 can activate AKT through its downstream effector, p21-activated kinase (PAK). Thus, we hypothesized that FAM134B activates AKT via the FAM134B-DDX3X-Rac1 signaling pathway. To test this hypothesis, we first assessed the impact of Rac1 on AKT phosphorylation in HCC. As anticipated, suppressing Rac1 expression with small interfering RNA (siRNA) reduced phosphorylated AKT levels in Huh7 and MHCC97-H cells (Fig. S3D). Moreover, inhibiting Rac1 expression negated the AKT phosphorylation induced by overexpression of both DDX3X and FAM134B (Fig. S3E, F). These results suggested that Rac1 mediates FAM134B’s activation of AKT signaling. To explore whether FAM134B promotes HCC progression through DDX3X expression, we established two cell lines: one with overexpression of FAM134B and reduced DDX3X expression (Hep3B), and another with reduced FAM134B and overexpression of DDX3X (MHCC97-H). Western blot confirmed the successful construction of these cell lines and demonstrated that FAM134B activated AKT signaling via DDX3X expression (Fig. 5A). CCK-8 assay (Fig. 5B), colony formation assay (Fig. 5C, D, Fig. S3G, H), and transwell assay (Fig. 5E–H, Fig. S3I) showed that the reduction of DDX3X abolished the FAM134B-mediated promotional effects, while DDX3X overexpression restored the impaired proliferation and metastasis abilities caused by FAM134B reduction. In vivo experiments also indicated that DDX3X overexpression partially recovered the proliferation and metastasis abilities impaired by FAM134B reduction (Fig. 5I–O). In conclusion, FAM134B promotes HCC progression through the FAM134B-DDX3X-Rac1-AKT signaling pathway.

### DDX3X promotes FAM134B transcription

**Fig. 6:**
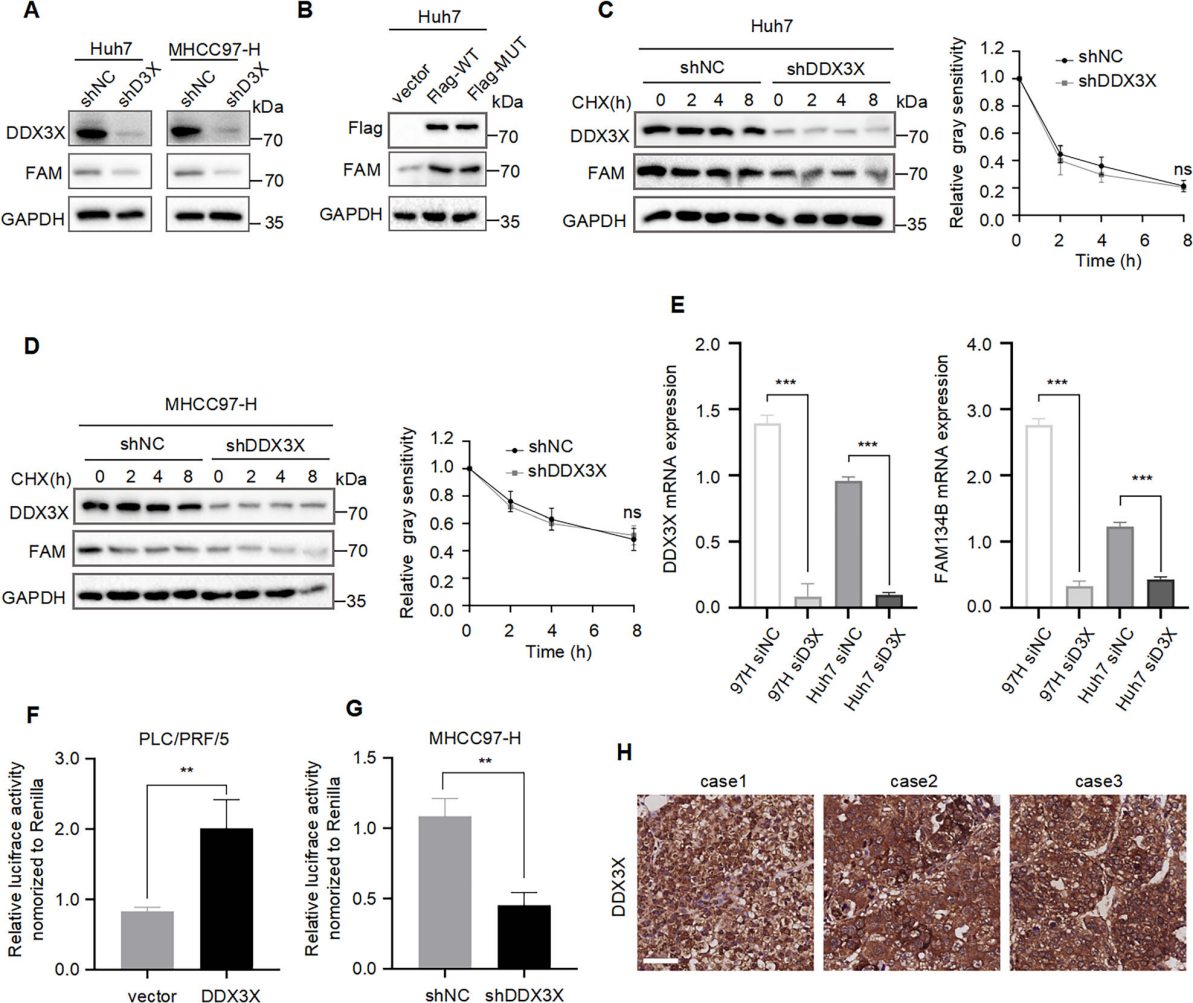
DDX3X promotes FAM134B transcription. **A** Western blot detected FAM134B protein level in Huh7 and MHCC97-H cells stably expressing shNC or shDDX3X. **B** MHCC97-H cells were transfected with vector, Flag-WT DDX3X, Flag-mutant DDX3X at the same concentration. Forty-eight hours later, cells were lysed with RIPA, followed by western blot analysis. **C** Huh7 cells stably expressing either shNC or shDDX3X were treated with CHX (20 μM) for 0, 2, 4, 8 h. The degradation kinetics of FAM134B was monitored over time by western blot. Data are mean ± SEM from three independent experiments, ns: not significant. **D** MHCC97-H cells stably expressing either shNC or shDDX3X were treated with CHX (20 μM) for 0, 2, 4, 8 h. The degradation kinetics of FAM134B was monitored over time by western blot. Data are mean ± SEM from three independent experiments, ns: not significant. **E** MHCC97-H and Huh7 cells were transfected with siNC or siDDX3X. Twenty-four hours later, total RNA was isolated using TRIzol reagent. RT-qPCR was then employed to assess the mRNA levels of DDX3X and FAM134B. Data are mean ± SEM from three independent experiments, ***P < 0.001. **F**, **G** Luciferase reporter assay detected the effect of DDX3X on FAM134B promoter activity. Data are mean ± SEM from three independent experiments, **P < 0.01. **H** The representative IHC images of DDX3X protein expression in HCC tissues. Scale bar, 40 μm.

When constructing rescue cell lines, we unexpectedly observed that DDX3X might influence the expression of the FAM134B protein (Fig. 5A). Consequently, we re-examined this regulatory relationship and sought to determine the mechanism by which DDX3X regulates FAM134B expression. First, we confirmed that the knockdown of DDX3X in MHCC97-H and Huh7 cell lines significantly reduced FAM134B protein levels, as demonstrated by western blot analysis (Fig. 6A). Second, DDX3X is renowned for its helicase activity. To explore this, we transfected MHCC97-H cells with both wild-type (WT) DDX3X and a helicase-deficient mutant (DDX3X^S382A,T384A^). The results indicated that both WT and mutant DDX3X could enhance FAM134B protein expression (Fig. 6B), suggesting that DDX3X’s regulation of FAM134B is independent of its helicase activity. Third, a half-life assay revealed that DDX3X downregulated FAM134B protein expression without affecting its stability (Fig. 6C, D). Furthermore, DDX3X is known to have transcriptional activation capabilities, potentially through interaction with transcription factors [16, 41, 42]. The knockdown of DDX3X led to a decrease in FAM134B mRNA levels in both MHCC97-H and Huh7 cell lines (Fig. 6E). Numerous studies have confirmed DDX3X’s ability to interact with transcription factors to promote gene transcription [16, 41, 42]. Therefore, we investigated whether DDX3X could enhance FAM134B transcription. A dual luciferase reporter assay confirmed that DDX3X indeed increased FAM134B promoter activity (Fig. 6F, G). Additionally, immunohistochemical (IHC) analysis of DDX3X expression in an HCC tissue microarray showed that DDX3X is localized to both the cytoplasm and the nucleus (Fig. 6H), supporting the reliability of DDX3X’s transcriptional regulation of FAM134B. In summary, DDX3X upregulates FAM134B expression through transcriptional activation. Furthermore, FAM134B and DDX3X appear to mutually regulate each other, forming a feedback loop.

### DDX3X is overexpressed in HCC and associated with FAM134B expression

**Fig. 7:**
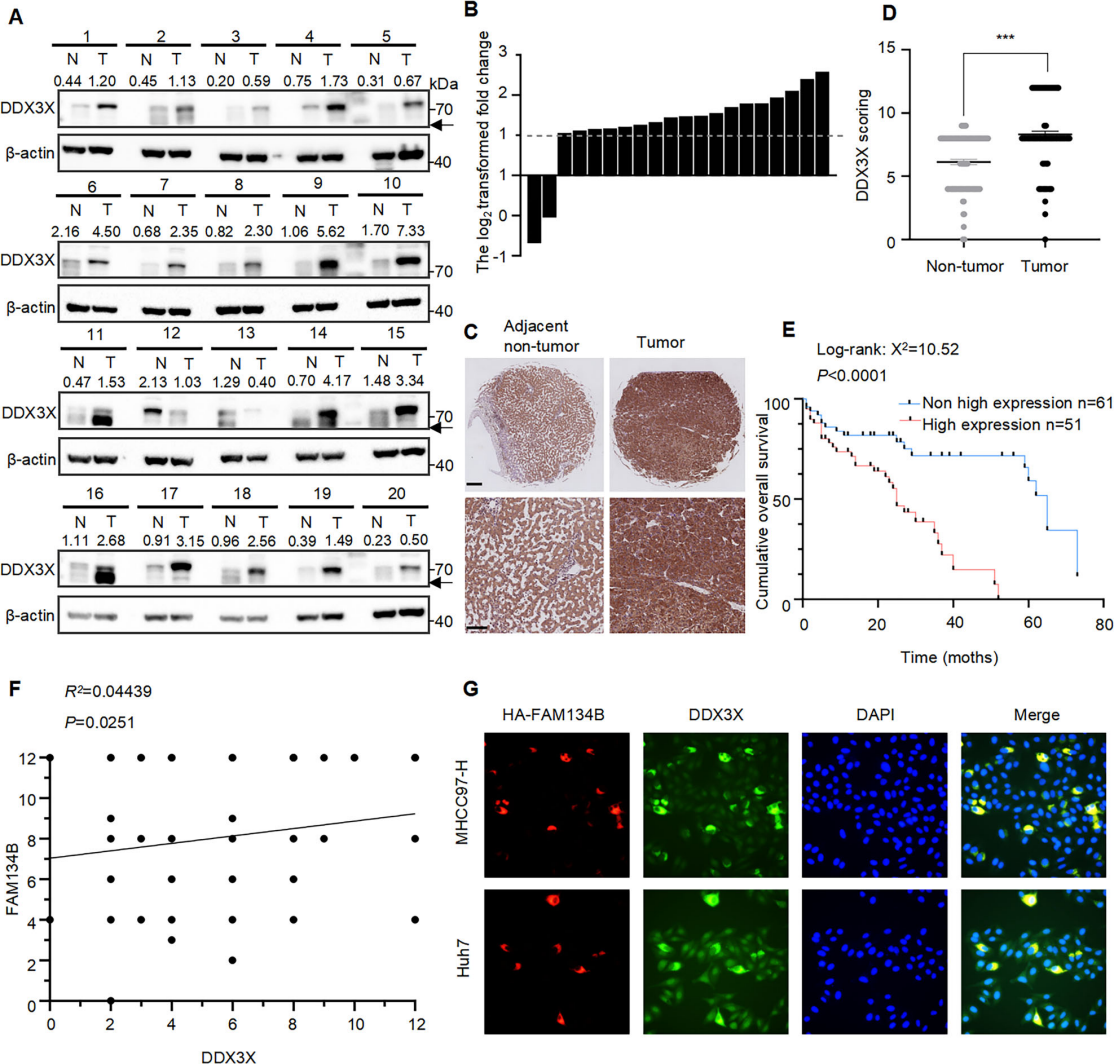
DDX3X is upregulated in HCC and associated with FAM134B expression. **A** Western blot detected the protein expression of DDX3X in 20 paired HCC tissues and adjacent non-tumor tissues, arrows indicated non-specific bands. The numbers indicated the relative amount of DDX3X signal compared to the first lane on the left, normalized to β-actin. **B** The log2 transformed fold change of DDX3X expression in tumor tissues and non-tumor tissues from (**A**). **C** The representative images of DDX3X expression in tissue microarray. Scale bar 4000 μm (top), 100 μm (bottom). **D** DDX3X scoring of paired HCC tissues and adjacent non-tumor tissues. Scale bar represents SEM, ***P < 0.001, *n* = 122. **E** Kaplan-Meier curves of the overall survival (OS) rates between groups with differential DDX3X expression. *P* < 0.0001, *n* = 112. **F** Pearson’s correlation analysis of FAM134B and DDX3X protein levels. *P* = 0.0251. **G** MHCC97-H and Huh7 cells were transfected with HA-FAM134B. Forty-eight hours later, cells were fixed and incubated with anti-HA and anti-DDX3X antibodies. The expression of HA-FAM134B and DDX3X were observed with fluorescence microscope.

To determine the protein expression levels of DDX3X in HCC, we compared its expression in 20 pairs of HCC tumor tissues to adjacent non-tumor tissues. Western blot analysis was conducted to measure DDX3X protein levels (Fig. 7A). The log2 transformed fold change of DDX3X in HCC revealed that overexpression of DDX3X defined as a >1-fold increase-was observed in 18 out of 20 (90%) pairs of primary HCC tumors compared to adjacent non-tumor tissues (Fig. 7B). Additionally, we assessed DDX3X expression in a tissue microarray comprising 122 pairs of HCC and adjacent non-tumor tissue samples with associated clinicopathological features (Fig. 7C, D). IHC results indicated that the average expression level of DDX3X was elevated in HCC tissues relative to adjacent non-tumor tissues (Fig. 7D). To explore the clinical significance of DDX3X expression in HCC, we analyzed the clinicopathological features of the aforementioned tissue microarray. Chi-square (and Fisher’s exact) test demonstrated an association between DDX3X expression and elevated serum alpha-fetoprotein levels, larger tumor size as well as advanced TNM stage (Table 3). Furthermore, Kaplan-Meier analysis revealed that patients with high DDX3X expression exhibited shorter overall survival (OS) compared to those with non-high DDX3X expression (Fig. 7E). Pearson correlation analysis showed a positive correlation between the expression levels of FAM134B [13] and DDX3X (Fig. 7F). IF demonstrated that in MHCC97-H and Huh7 cells, cells with high expression of HA-FAM134B also exhibited higher DDX3X expression levels (Fig. 7G). In conclusion, our findings confirm that DDX3X is overexpressed in HCC and is correlated with FAM134B expression.

**Table 3:** Clinicopathological correlation of DDX3X expression in HCC.

Characteristics		Total	Non-high expression of DDX3X	High expression of DDX3X	*P*
Gender	Male	103	37	66	0.7987
	Female	19	6	13	
Age	≤55 years	77	28	49	0.8448
	>55 years	45	15	30	
Serum Alpha-fetoprotein	<400 ng/ml	65	30	35	**0.0019**
	≥400 ng/ml	57	13	44	
Tumor size^a^	<5 cm	48	23	25	**0.0212**
	≥5 cm	74	20	54	
Cirrhosis	Absent	33	15	18	0.2004
	Present	89	28	61	
Tumor encapsulation	Absent	56	24	32	0.1293
	Present	66	19	47	
Pathological vascular invasion	Absent	74	28	46	0.5613
	Present	48	15	33	
Cancer recurrence	Absent	60	25	35	0.1850
	Present	62	18	44	
Differentiation grade	High grade	29	13	16	0.2668
	Low grade	93	30	63	
TNM	Ⅰ-Ⅱ	87	37	50	**0.0112**
	Ⅲ-Ⅵ	35	6	29	
Portal vein tumor thrombus	Absent	101	33	68	0.2155
	Present	21	10	11	

^a^Tumor size was measured by the length of the largest tumor nodule.

*P-*values of the characteristics with statistical significant were bolded.

### Synergistic effect of treatment with DDX3X inhibitor RK-33 and knockdown of FAM134B

**Fig. 8:**
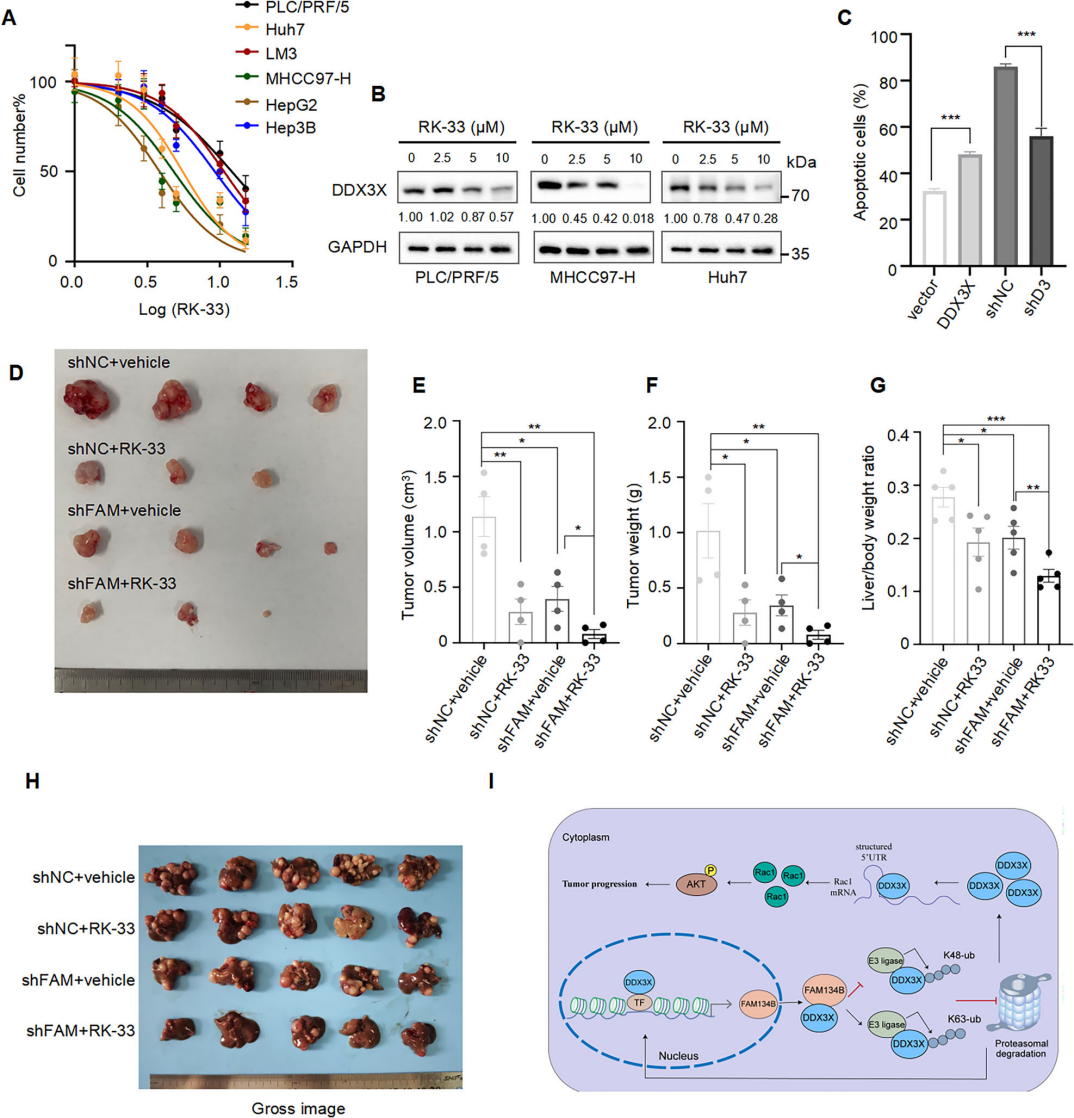
Synergistic effect of treatment with DDX3X inhibitor RK-33 and knockdown of FAM134B. **A** MHCC97-H, HepG2, Huh7, Hep3B, LM3, PLC/PRF/5 cells were treated with RK-33 at varying concentrations for 24 hours. The cell survival rate was then determined using the CCK-8 assay. Scale bar represents SEM, *n* = 5. **B** PLC/PRF/5, MHCC97-H, Huh7 cells were treated the RK-33 at concentration of 0, 5, 10, 15 μM for 24 h. Western blot detected DDX3X protein level. The numbers indicated the relative amount of DDX3X signal compared to the first lane on the left, normalized to GAPDH. **C** Statistical chart of apoptotic cells from the indicated cell lines. Scale bar represents SEM, *n* = 3, ***P < 0.001. **D**−**F** The gross image (**D**), tumor volume (**E**), tumor weight (**F**) of subcutaneous tumor model from the indicated group, *n* = 4, scale bar represents SEM, *P < 0.5, **P < 0.01. **G** The Liver/body weight ratio from HTVi model, *n* = 5, scale bar represents SEM, *P < 0.5, **P < 0.01, ***P < 0.001. **H** The representative gross images of HTVi model. **I** Graphical summary of the study. The interaction between FAM134B and DDX3X inhibits DDX3X K48-linked polyubiquitination and promotes its K63-linked polyubiquitination, thereby stabilizing the expression of the DDX3X protein. Subsequently, DDX3X activates the AKT signaling pathway by facilitating the translation of Rac1. Additionally, DDX3X enhances the transcription of FAM134B, suggesting the existence of a positive feedback loop between these two proteins.

Finally, we evaluated the therapeutic effect of the DDX3X inhibitor RK-33 [43] in combination with FAM134B reduction in HCC treatment. RK-33 is a small molecule that binds to the ATPase domain of DDX3X, inhibiting its helicase unwinding activity. The CCK-8 assay revealed that the HepG2 (IC50 = 3.846 μM), Huh7 (IC50 = 5.541 μM), and MHCC97-H (IC50 = 4.809 μM) cell lines, which exhibited high levels of DDX3X, demonstrated a significantly greater sensitivity to RK-33 treatment compared to the PLC/PRF/5 (IC50 = 12.05 μM), Hep3B (IC50 = 9.214 μM), and LM3 (IC50 = 10.67 μM) cell lines, which exhibited low levels of DDX3X (Fig. 8A). Consistently, western blot showed that RK-33 efficiently decreased DDX3X protein levels in MHCC97-H and Huh7 cell lines (Fig. 8B). Subsequently, we investigated the impact of RK-33 on apoptosis in HCC cells. Flow cytometry analysis revealed that PLC/PRF/5 cells overexpressing DDX3X were more susceptible to RK-33 treatment than control counterparts. Conversely, MHCC97-H cells with DDX3X knockdown exhibited greater stability under RK-33 treatment compared to control cells (Fig. 8C and Fig. S4A). Given the mutual promotion of expression between FAM134B and DDX3X, we hypothesized that the combination of DDX3X inhibitor and FAM134B knockdown might enhance the therapeutic effect in HCC cells. We divided sixteen nude mice into four groups, with two groups injected with FAM134B-knockdown cells and the other two with scramble control cells into the dorsal flank. When tumor volumes reached 40–60 mm^3^, vehicle or RK-33 (20 mg/kg) was administered twice weekly for 5 weeks. As shown in Fig. 8D–F, tumors treated with vehicle were generally heavier and larger than those treated with RK-33 (Fig. 8D–F). Notably, the weight and volume of tumors treated with FAM134B knockdown plus RK-33 were significantly lighter and smaller than those of the other groups (Fig. 8D–F). Western blot indicated that RK-33 had great effect on the expression levels of DDX3X (Fig. S4B). Moreover, the combination of FAM134B knockdown and RK-33 treatment exhibited the most pronounced inhibitory effect on AKT phosphorylation in vivo (Fig. S4B). Additionally, we established a hypertension teil vein injection model (HTVi) by injecting pT3-myr-akt, pT3-c-myc, and pcDNA3.1/px458-shFAM134B. Two weeks later, mice were treated with vehicle or RK-33 (20 mg/kg) three times per week for 2 weeks. The results showed that the liver/body weight ratio of the FAM134B knockdown plus RK-33 group was significantly smaller than that of the other groups (Fig. 8G, H). Western blot analysis confirmed the efficiency of px458-shFAM134B plasmid. Consistent with the subcutaneous tumor model, FAM134B knockdown combined with RK-33 had the strongest effect on AKT phosphorylation inhibition (Fig. S4C). Collectively, this evidence demonstrates a synergistic therapeutic effect when using the DDX3X inhibitor RK-33 in conjunction with FAM134B knockdown.

## Discussion

Our previous research established that FAM134B promotes HCC progression by activating AKT phosphorylation. However, the precise mechanism of AKT activation by FAM134B remains to be elucidated. In this study, we sought to identify the molecular mechanism underlying AKT activation by FAM134B, focusing on protein-protein interactions. We utilized immunoprecipitation followed by IP-MS to identify potential binding partners of FAM134B. Notably, DDX3X was identified, a protein known to be involved in AKT activation. Subsequent experiments confirmed that FAM134B interacts with DDX3X, inhibiting DDX3X K48-polyubiquitination and enhancing K63-polyubiquitination. Recent studies have reported various regulatory mechanisms of DDX3X expression, including post-transcriptional and post-translational regulations [33, 44, 45]. Post-translationally, E3 ligase RNF39 ubiquitinates DDX3X at lysine residues 55, 138, and 162, leading to K48-linked ubiquitination and subsequent proteasomal degradation of DDX3X [46]. Conversely, SIRT7 deacetylates DDX3X at lysine 55, reducing its total ubiquitination and thereby inhibiting DDX3X proteasomal degradation [33]. Our research corroborated the involvement of the ubiquitin-proteasome degradation pathway in DDX3X regulation. Interestingly, we discovered that FAM134B stabilizes DDX3X protein levels by decreasing K48-polyubiquitination and increasing K63-polyubiquitination, with K63-polyubiquitination being the predominant regulatory mechanism. While K63-polyubiquitination is typically associated with non-proteolytic functions, emerging evidence suggests it also plays a role in stabilizing protein expression. However, a separate study reported that E3 ligase WWP2 increases DDX3X K63-polyubiquitination, promoting its proteasomal degradation in Type 2 diabetes [47]. The contrasting results regarding the effects of K63-polyubiquitination on DDX3X may be attributed to modifications of different lysine residues or the influence of disease context.

DDX3X has been demonstrated to be highly expressed in numerous malignancies and contributes to oncogenic processes in tumor progression. Our study corroborated these findings, confirming that DDX3X promotes both proliferation and metastasis of hepatocellular carcinoma (HCC) in both in vivo and in vitro models. Additionally, our research established a correlation between high DDX3X expression and poor prognosis in HCC, which is in line with previous reports. DDX3X is known to activate AKT phosphorylation through the promotion of Rac1 translation and the inhibition of PTEN signaling. In our study, we confirmed that DDX3X was positively correlated with p-AKT levels in PLC/PRF/5, MHCC97-H and Huh7 cell lines, which had high basal activity. There are many factors that affect AKT activity, such as PIK3CA mutation, PTEN function and the activity of upstream receptor tyrosine kinases. There is a missense mutation in the PTEN gene in Huh7 cell line [48], which might lead to the activity of PI3K/AKT signaling pathway. Persistent activation of upstream receptor tyrosine kinases, particularly IGF autocrine loops, further amplifies the pathway. Huh7 is a well-characterized cell line with high IGF-2 expression [49]. Mutations in PTEN or PIK3CA have not been reported in the other two cell lines. Even though, the three cell lines also expresses various oncogenes or carries somatic mutations in multiple other genes, which may be associated with the activation of the AKT pathway. Our new data show that DDX3X continues to modulate AKT even under these constitutively active conditions, indicated that DDX3X operates downstream of-or parallel to-the underlying genomic aberrations that sustain AKT signaling. Mechanically, our study confirmed that DDX3X activates AKT Ser473 phosphorylation via the regulation of Rac1 in HCC, without affecting PTEN expression. This led us to hypothesize that FAM134B activates AKT phosphorylation through DDX3X-mediated Rac1 translation, a hypothesis that was substantiated in subsequent experiments.

In recent years, the regulatory mechanisms of FAM134B expression have been increasingly elucidated. Transcriptional regulation occurs through Microphthalmia-associated transcription factors (MiTF/TFE) [50], which bind directly to the CLEAR site located in the third intron of the FAM134B gene, thereby promoting FAM134B expression [50]. Post-translationally, USP20 has been reported to deubiquitinate FAM134B, leading to an increase in its protein expression [51]. Furthermore, DDX3X is known to bind with transcription factors SP1 and YY1 to promote the transcription of downstream genes, including KRAS, P21, WNT, and so on [41, 52, 53]. Our results indicate that DDX3X upregulates FAM134B protein levels by enhancing transcriptional activity, revealing a novel mechanism for the transcriptional regulation of FAM134B. However, further experiments are required to identify the specific transcription factors that interact with DDX3X and promote FAM134B transcription.

RK-33 is a small molecule that binds to the ATPase domain of DDX3X [43], thereby inhibiting its helicase unwinding activity, which is crucial in tumor biology. Numerous studies have investigated the inhibitory effects of RK-33, leveraging DDX3X’s role in cell cycle arrest and the DNA repair pathway [28, 29, 33, 43, 45, 54]. Consequently, there has been a focus on the therapeutic potential of combining RK-33 with chemotherapy drugs in cancer treatment. In HCC, DDX3X-mediated NLRP3 inflammasome assembly has been implicated in sorafenib resistance. The combination of RK-33 with sorafenib has been shown to overcome this chemoresistance [33]. In our study, we discovered a mutual promotion of expression between DDX3X and FAM134B. Consequently, we explored the potential of combining FAM134B expression interference with RK-33 treatment to enhance therapeutic outcomes. However, it is worth noting that, to date, no specific inhibitor for FAM134B has been identified, which limits the exploration of such combination therapies.

## Conclusion

In summary, we demonstrated that FAM134B activated AKT signaling via DDX3X-Rac1-AKT axis in HCC. Mechanistically, FAM134B inhibited DDX3X proteasomal degradation. The upregulated DDX3X activated AKT signaling via promoting Rac1 translation. Moreover, DDX3X enhanced the transcription of FAM134B, suggesting a positive feedback loop. Combination of DDX3X inhibitor RK-33 with FAM134B knockdown effectively inhibited HCC progression in vivo.

## Materials and methods

### Cell culture

The China Center for Type Culture Collection (Wuhan, China) provided human HCC cell lines, including HLF, Hep3B, LM3, MHCC97H, HepG2 and PLC/PRF/5. The human embryonic kidney cell line 293 T (HEK-293T) was purchased from the Shanghai Branch Cell Bank of the Chinese Academy of Sciences (Shanghai, China). All cell lines were recently authenticated by STR profiling and tested for mycoplasma contamination. Cells were cultured in Dulbecco’s modified Eagle’s medium (DMEM, Sigma) supplemented with 10% fetal bovine serum (Hyclone) and maintained at 37 °C in a humid incubator with 5% CO_2_.

### Plasmids and construction of stable cell lines

E. coli strains carrying the DDX3X and FAM134B genes were obtained from WZ Biosciences Inc. (Shandong, China). The cDNAs for DDX3X and FAM134B were subcloned into the pcDNA3.1(+) expression vector (Sigma-Aldrich). HA-tag, Myc-tag, and Flag-tag sequences were incorporated using corresponding primers. Additionally, the FAM134B promoter sequence was cloned into the pGL4.17 vector. Purinomycin (Solarbio Life Science) was utilized to generate and evaluate a stable FAM134B and DDX3X overexpressing and lines. The FAM134B and DDX3X small hairpin RNA (shRNA) was donated by Sigma Corporation. Following the manufacturer’s instructions, stable FAM134B-knockdown and DDX3X knockdown cells were created using a lentiviral vector. ShRNA sequences: ShFAM134B-1 (5’-CTACTGTTACTGTGTGCATTT). ShFAM134B-2 (5’-GCAGCTATCAAAGACCAGTTACTCGAG-3’). ShDDX3X (5’-CGGAGTGATTACGATGGCATT-3’).

### siRNAs

SiRNA(5′-AGACGGAGCTGTAGGTAAA-3′) targets the coding region of homo sapiens Rac1. SiRNA (5’-CGGAGTGATTACGATGGCATT-3’) targets the coding region of homo sapiens DDX3X.

### Patient samples

The clinical samples utilized in this study were provided by Tongji Hospital of Huazhong University of Science and Technology (HUST) in Wuhan, China, and the study protocol received approval from the HUST Ethics Committee at Tongji Hospital. All patients were informed consent.

### Animal models

For subcutaneous tumor model and lung metastasis model, the animals used were 4-week-old, male, BALB/c nude mice. For subcutaneous tumor model, each mouse was injected with 1,000,000 cells/100 µL. Four weeks later, tumors were removed, fixed, weighed, photographed and stored. For the lung metastasis model, each mouse was injected with 1,000,000 cells/100 µL in the tail vein. Four weeks later, lungs were removed, fixed, photographed and stored. For HTVi model, C57BL/6 J mice were purchased from Charles River Laboratories. At 6−8 weeks (body weight 20−22 g), each mouse was injected with pT3-myr-akt (20 μg)/pT3-c-myc (20 μg)/pcDNA3.1 (20 μg)/ px458-shFAM134B (20 μg) and sleeping beauty transposase plasmids (1/25 of the total plasmid mass) through the tail vein. Treatment started after 2 weeks of injection. RK-33 (S8246, Selleck, China) were solved in 10% DMSO (472301, Sigma-Aldrich), 40% PEG300 (S6704, Selleck, China), 5% Tween 80 (S6702, Selleck, China) and 40% bi-distilled water. All animal care and research were conducted in accordance with the guidelines for the care and use of experimental animals of the National Institutes of Health and approved by the ethics committee of HUST Ethics Committee at Tongji Hospital.

### CCK-8 assay

HCC cells were inoculated into 96-well plates at 1000 cells per well. Approximately 6–8 h were allowed for the cells to attach to the wall, that is, Day 0. CCK-8 Cell Counting Kit (A311-01, Vazyme) was used to test the OD (450 nm) value over 5 days.

### Transwell assay

Cells were seeded into upper chambers as follows: 250,000 cells for migration assay and 500,000 cells for invasion assay. For invasion assay, diluted matrigel (diluted with DMEM at 1:4) was added into the upper chamber before cells inoculation. Complete medium was added into the lower chambers. After 24 ~ 48 h, upper chambers were washed with PBS, then fixed with 4% paraformaldehyde for 15 min and stained with crystal violet for 15 min. Cells were counted by Image J.

### Would healing assay

Cells were seeded into a six-well plate. After the cells were fully walled, a pipette was used to scrape across the confluent cell layer to form a linear wound. Then, complete medium was replaced with DMEM. Photographs were taken at 0, 24 and 48 h to observe cell migration and the degree of healing was calculated using Image J.

### Colony formation assay

HCC cells were inoculated into six-well plates with three replicate wells at 1000 cells per well. Two weeks later (replacing the complete medium every 3 days), cells were fixed with 4% paraformaldehyde, then stained with crystal violet, colonies were counted by image J.

### Soft agar assay

The bottom agar was prepared as follows: 50 µL of 0.6% agar in DMEM, containing 10% FBS, was pre-warmed to 37 °C, transferred into 24-well plates, and chilled at room temperature until the bottom agar solidifies. Cells were diluted with DMEM and mixed into 0.6% agar at a ratio of 1:2, to obtain a final concentration of 0.4% agar containing cells. For each well, 200 µL of the 0.4% agar containing cells was layered onto the 0.6% bottom agar in the 24-well plates and chilled at room temperature until agar solidifies. Complete DMEM was added to the cell agar (replaced every three days). Two weeks later, images were taken. Colonies were counted by Image J.

### Luciferase reporter assay

Cells were seeded into a 24-well plate at 40% density, then transfected with 200 ng pGL4.17 plasmid and 4 ng pRL-TK-Renilla-luciferase plasmid. 48 h later, cells were collected with Passive Lysis Buffer (Promega) according to the instructions. The next steps were performed according to the protocol. The luciferase activity was measured by GloMax 20/20 luminometer (Promega). The relative luciferase activity was determined based on the ratio of firefly luciferase to Renilla luciferase.

### RNA extraction and quantitative real-time PCR (RT-qPCR)

Total RNAs were extracted from cells using the FastPure Cell/Tissue Total RNA Isolation Kit V2 (RC112-01, Vazyme). Reverse transcription was carried out with the reverse transcription kit (R233-01, Vazyme). For qPCR, the Universal SYBR qPCR Master Mix kit (Vazyme, Q711-02) was employed.

### Western blot

Western blot was performed according to the conventional procedure. Anti-DDX3X antibody (1:1000, 11115-1-AP), Anti-FAM134B antibody (1:1000, 21537-1-AP), Anti-GAPDH antibody (1:5000, 10494-1-AP), Anti-Rac1 antibody (1:500, 24072-1-AP) were purchased from Proteintech. Anti-AKT antibody (1:1000, 9272), Anti-p-AKT Ser473 antibody (1:1000, 4060) were purchased from CST. Anti-Flag antibody (1:3000, F1804), Anti-HA antibody (1:2000, H9658) were purchased from Sigma. Anti-Myc antibody (1:5000, AE070) was purchased from ABclonal.

### Co-immunoprecipitation (co-IP)

Cells were lysed with IP-lysis followed by incubating with antibodies over night at 4 °C. The lysis then incubated with washed protein A/G magnetic beads (Biolinkedin, L-1004) for 2 ~ 3 h at 4 °C. Washing magnetic beads with TBST for 5 times. Discarding TBST, adding 50 ~ 100 µL 1 × loading buffer, and incubating the specimens in boiling water for 10 min. Storing the specimens at -80 °C. Anti-DDX3X antibody (1:200, 11115-1-AP) was purchased from Proteintech. Anti-FAM134B antibody (1:200, E8Y9R) was purchased from CST. Anti-Flag antibody (1:500, F1804), Anti-HA antibody (1:500, H9658) were purchased from Sigma. Anti-Myc antibody (1:500, AE070) was purchased from ABclonal.

### Immunofluorescence (IF)

Cells were fixed with 4% paraformaldehyde for 15 min, then permeabilized with 1% Triton X-100 for 15 ~ 30 min. 5% bovine serum albumin blocked cells for 30 min at room temperature. Cells then incubated with primary antibodies at 4 °C overnight. The next day, incubated cells with the second antibodies at room temperature for 1 h. Images were taken by Zeiss LSM900. Anti-DDX3X antibody (1:200, 11115-1-AP) was purchased from Proteintech. Anti-FAM134B antibody (1:200, E8Y9R) was purchased from CST.

### Statistical analysis

All the data are presented as the means ± SEMs. Differences between the experimental and control groups were calculated using Student’s *t*-test or one-way ANOVA with Tukey’s multiple comparisons test (GraphPad Prism). Significant *P-*values are *P < 0.05, **P < 0.01, and ***P < 0.001.

## Supplementary information

Supplementary Meterials

## Data Availability

The datasets generated and/or analysed during the current study are available from the corresponding authors on reasonable request.
