# Supplementary material for: Targeting FAM134B-DDX3X axis inhibiting AKT signaling in hepatocellular carcinoma
Source: Cell Death Dis. 2025 Nov 6;16(1):797. doi: 10.1038/s41419-025-08080-3 (PMC12592434; doi:10.1038/s41419-025-08080-3)
Supplement: Supplementary file 1 — Supplementary Meterials [file 41419_2025_8080_MOESM1_ESM.docx]

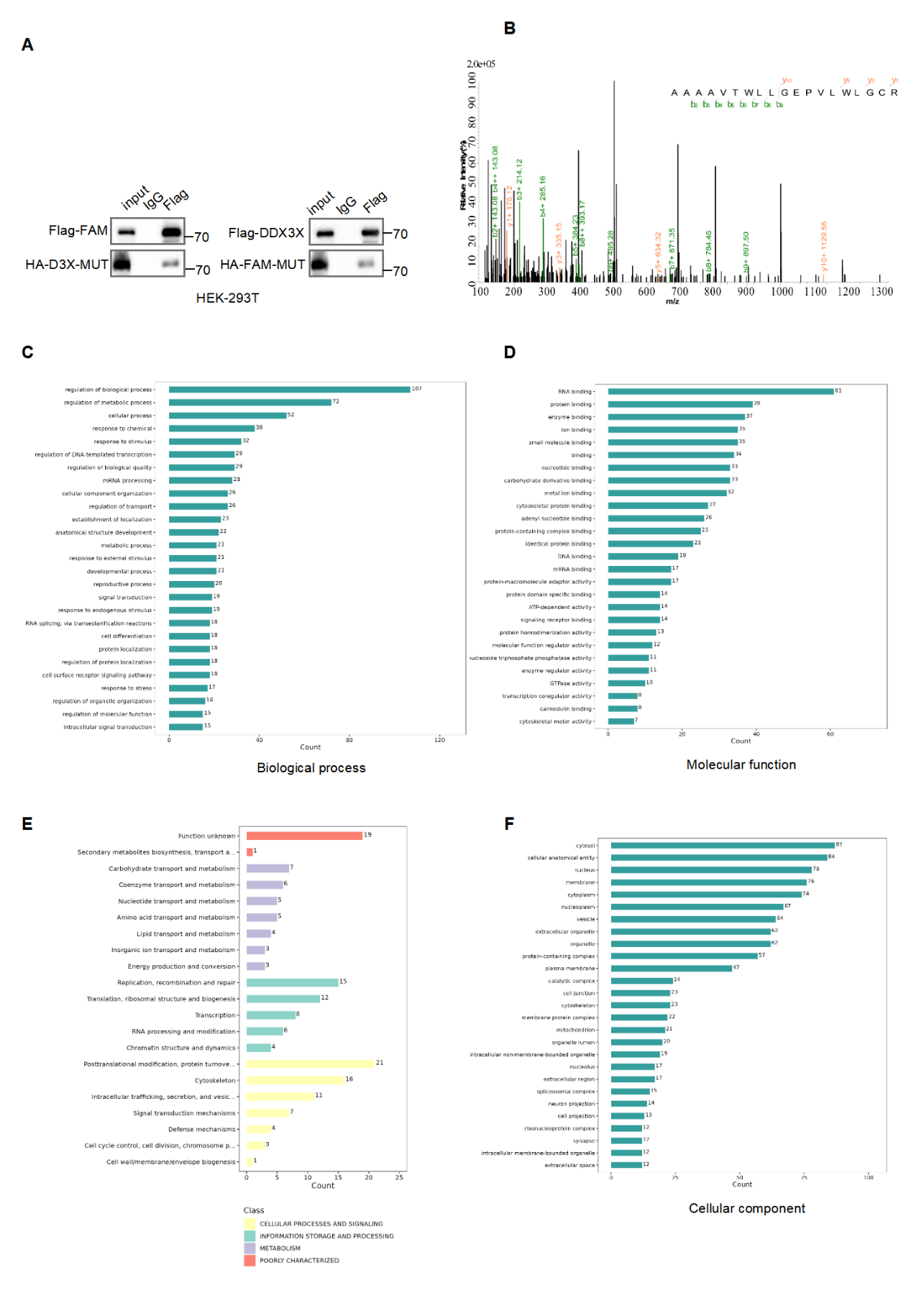
Figure S1 FAM134B interacts with DDX3X.

(A) Flag-FAM134B and HA-DDX3X-MUT, Flag-DDX3X and HA-FAM134B-MUT were transfected into HEK-293T cells. Forty-eight hours later, cells were lysed with IP-lysis, then immunoprecipitated with control IgG, anti-Flag or anti-HA antibodies. Western blot detected the input and immunoprecipitates. (B) Peptides of FAM134B identified by DDX3X IP-MS. (C and D) Gene Ontology (GO) analysis of DDX3X binding proteins on biological process (C) and molecular functions. (E) Clusters of Orthologous Groups of proteins (COG) analysis of DDX3X binding proteins. (F) GO analysis of DDX3X binding proteins on cellular component.


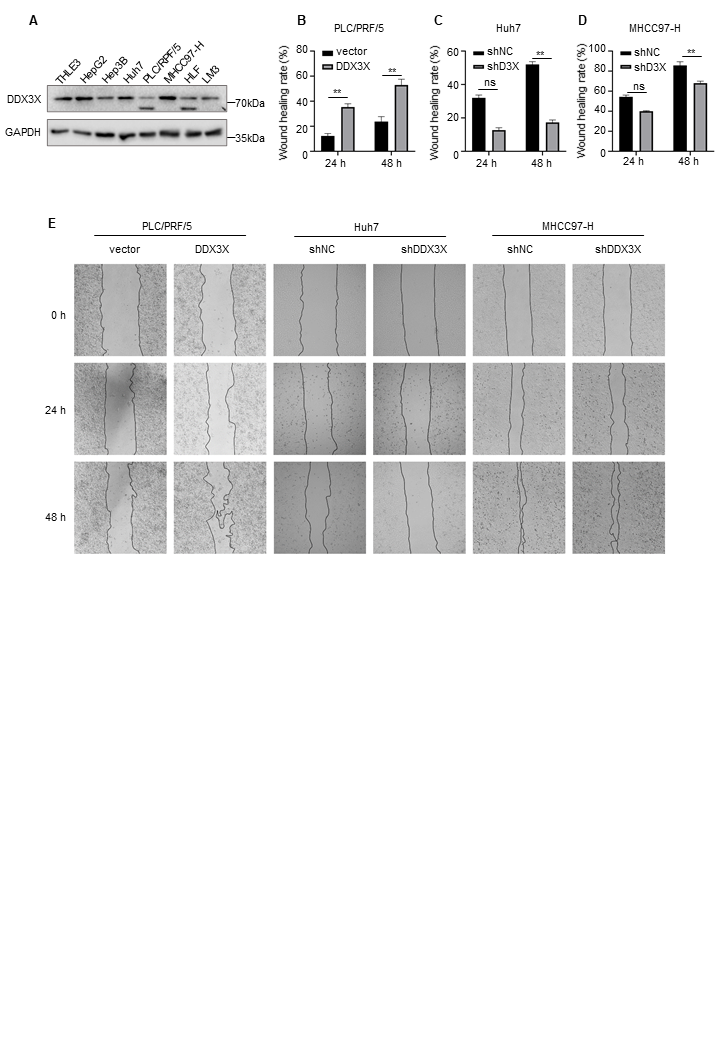


Figure S2 DDX3X promotes HCC cells migration *in vitro.*

(A) Western blot detected the protein expression of DDX3X in HCC cell lines. (B-E) Wound healing assay detected the impact of DDX3X on HCC cell migration. Scale bar represents SEM, n=3, ns: not significant, ***P*<0.01.


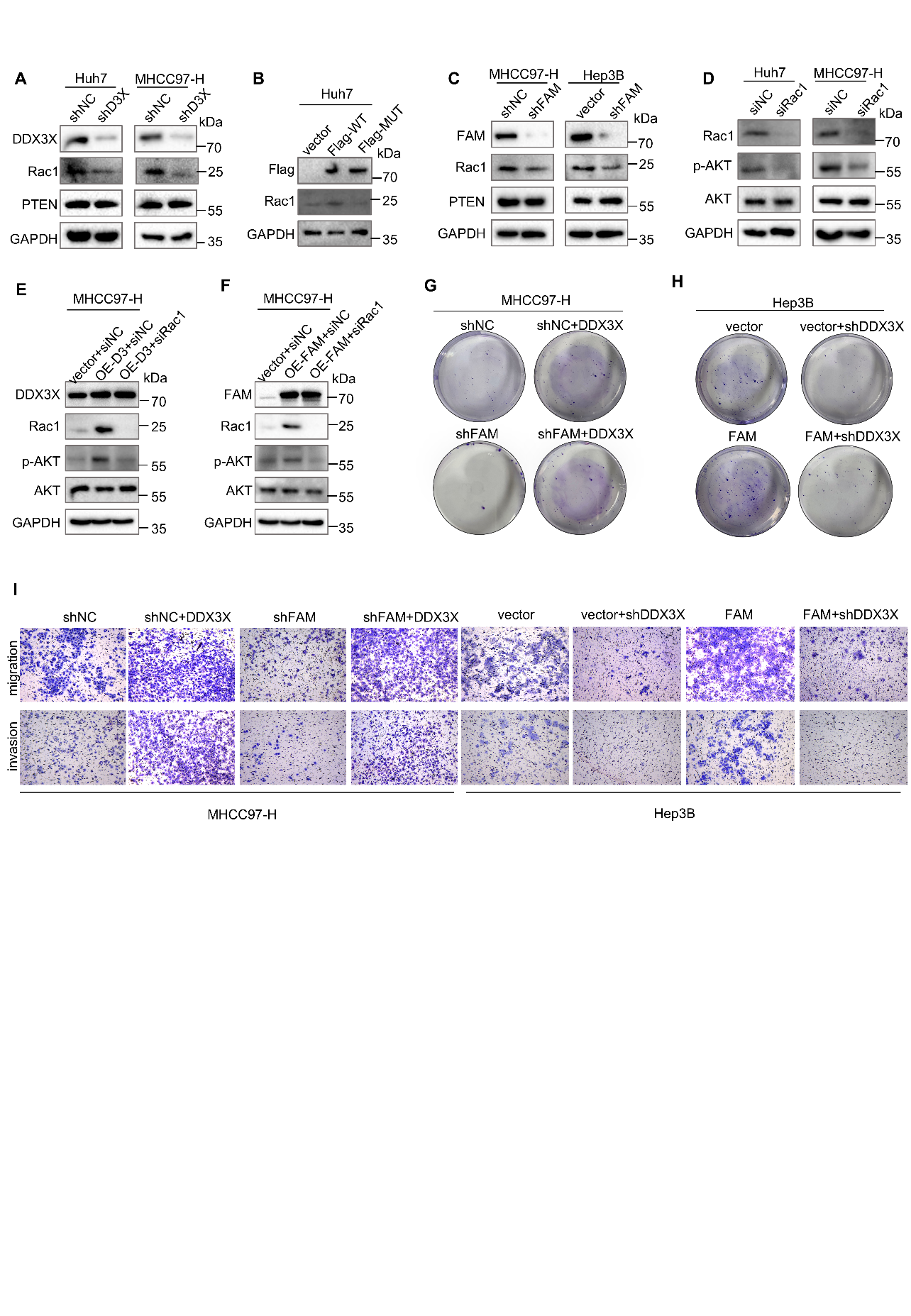
Figure S3 FAM134B-DDX3X-Rac1-AKT axis in HCC

(A) Western blot investigated the expression of Rac1 and PTEN in Huh7 and MHCC97-H cells stably expressing either shNC or shDDX3X. (B) Huh7 cells were transfected with vector, Flag-WT DDX3X, Flag-mutant DDX3X at the same concentration. Forty-eight hours later, western blot detected the expression of Rac1. (C) Western blot analysis investigated the expression of Rac1 and PTEN in Huh7 and MHCC97-H cells stably expressing either shNC or shFAM134B-1. (D) Huh7 and MHCC97-H cells were transfected with either siNC or siRac1. Forty-eight hours later, western blot was employed to detect p-AKT (Ser473) level. (E) MHCC97-H cells were transfected with vector+siNC, Flag-DDX3X+siNC, Flag-DDX3X+siRac1. Forty-eight hours later, western blot was employed to detect the expression of Rac1 and p-AKT (Ser473). (F) MHCC97-H cells were transfected with vector+siNC, Flag-FAM134B+siNC, Flag-FAM134B+siRac1. Forty-eight hours later, western blot was employed to detect the expression of Rac1 and p-AKT (Ser473). (G and H) Representative images of colony formation assay from indicated cell lines. (I) Representative images of Transwell assay form indicated cell lines.


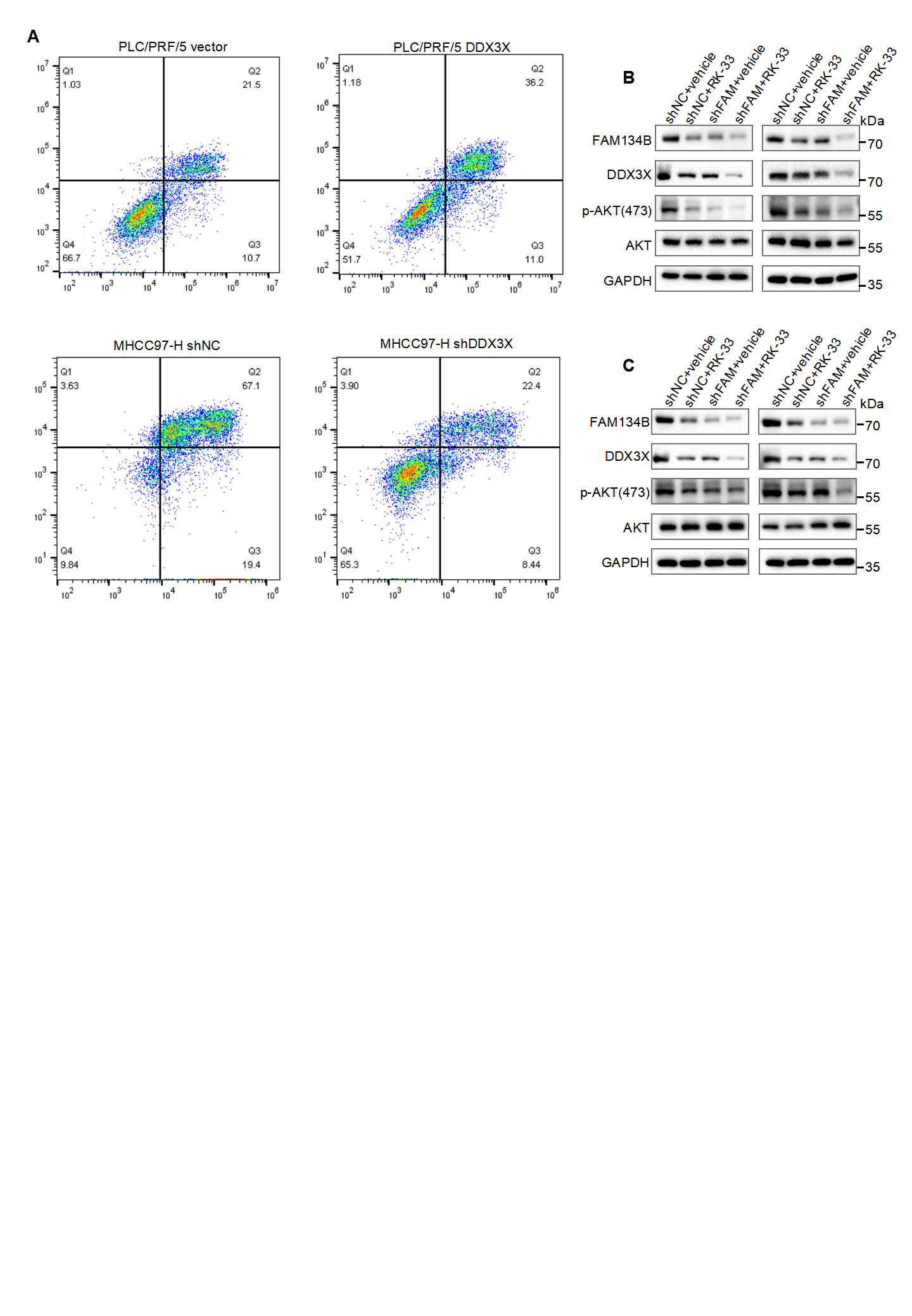


Figure S4 Synergistic effect of treatment with DDX3X inhibitor RK-33 and knockdown of FAM134B.

(A) PLC/PRF/5 cells stably expressing either vector or DDX3X and MHCC97-H cells stably expressing either shNC or shDDX3X were treated with RK-33 (PLC/PRF/5 12 μM, MHCC97-H 5 μM). Twenty-four hours later, flow cytometry detected cell apoptosis. (B and C) Western blot detected the protein level of FAM134B, DDX3X, p-AKT(Ser473), AKT and GAPDH from subcutaneous tumor model (B) and HTVi model (C).
